# Immediate reconstruction using a modified inframammary adipofascial flap after partial mastectomy

**DOI:** 10.1007/s00595-012-0390-7

**Published:** 2012-11-01

**Authors:** Yuko Kijima, Heiji Yoshinaka, Munetsugu Hirata, Tadao Mizoguchi, Sumiya Ishigami, Hideo Arima, Akihiro Nakajo, Shinichi Ueno, Shoji Natsugoe

**Affiliations:** Department of Digestive Surgery, Breast and Thyroid Surgery, Kagoshima University Graduate School of Medical and Dental Sciences, 8-35-1, Sakuragaoka, Kagoshima, 890-8520 Japan

**Keywords:** Breast cancer, Breast conservative therapy, Oncoplastic surgery, Immediate reconstruction, Modified inframammary adipofascial flap, Inframammary adipofascial flap

## Abstract

Breast conservative therapy (BCT) as treatment for early breast cancer usually ensures local control and acceptable cosmetic results. We describe a new technique of using an inframammary adipofascial flap to reconstruct defects caused by lower-pole partial mastectomy, which achieved excellent results (Kijima et al. in Am J Surg 193:789–91 (1); Sakai et al. in Ann Plast Surg 29(2):173–7, 2; Ogawa Am J Surg 193:514–8, 3). We developed this procedure as an oncoplastic technique for a Japanese woman with a similar defect without ptosis. After partial mastectomy, the superior half of the flap is harvested via an initial incision along the inframammary line, and the inferior half is harvested via an additional incision along the caudal edge of the flap, to produce a crescent of de-epithelialized skin. A tongue-shaped flap containing the crescent of de-epithelialized skin, subcutaneous fat, and the fascia of the vertical rectus abdominis muscle is then rotated upwards, gathered, and inserted into the breast defect.

## Introduction

BCT has become the standard treatment for breast cancer as it achieves local control with acceptable cosmetic results [4]. However, an insufficient resection margin may result in local recurrence if too much attention is paid to cosmesis. Immediate reconstruction after BCT has thus become popular for early stage breast cancer [5, 6]. We previously reported our early experience of an oncoplastic technique whereby an inframammary adipofascial flap is used to repair partial defects in the lower part of the breast in Japanese women [1]. This technique is considered more useful for breast surgeons than for plastic surgeons to repair breast defects immediately and prevent intra- or postoperative complications; however, it can be difficult to harvest sufficient flap material via an incision close to the cancer lesion without special devices because the posterior edge of the flap is so far from the incision line. Thus, we describe a modified technique for reconstructing defects in the lower part of the breast in patients with early breast cancer, using a modified inframammary adipofascial flap (an inframammary adipofascial flap with dermis).

### Diagnosis

**Fig. 1:**
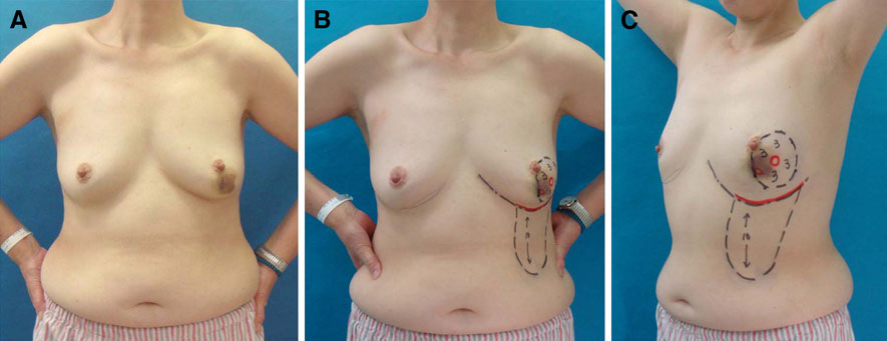
Case 1: preoperative markings of the area to be resected in a 53-year-old patient with a T1 tumor in the lower-outer quadrant of the left breast. **a** A *purple spot* formed after core needle biopsy (CNB). Her breasts were not ptotic. **b**, **c** The cancer lesion and the scar in the 6 o’clock position left by the CNB are circled in *red*. The *incision line is drawn in red* along the inframammary line

Mammography, ultrasonography, computed tomography, histological examination of a core needle biopsy (CNB) sample, bone scintigraphy, and magnetic resonance imaging were all performed preoperatively. The patient’s lesion was found to contain an invasive component and to be restricted to the lower-outer pole of the breast. The patient did not suffer from systemic disease or distant metastasis, her breasts were not ptotic, and the inframammary line could be distinguished from the anterior view in both the ‘arms up’ and ‘arms down’ positions (Fig. 1). Informed consent was obtained from the patient prior to oncoplastic surgery. She had a scar from the CNB at the 6 o’clock position of her left breast.

### Technique

After obtaining informed consent from the patient, a curved incision is made along the inframammary line to remove the lower area of the breast, including the scar from the CNB. The tumor is removed with gross margins of 2–3 cm, via a cylindrical resection, resulting in the fascia of the pectoral muscle also being removed (Fig. 1). Intraoperative pathological examinations of several surgical margins are then performed and, provided they are cancer-free, the reconstructive procedure can begin. Sentinel lymph node (SLN) biopsy using the RI method is performed via another incision in the axillary area to ensure that the resected SLN is negative for cancer metastasis.

**Fig. 2:**
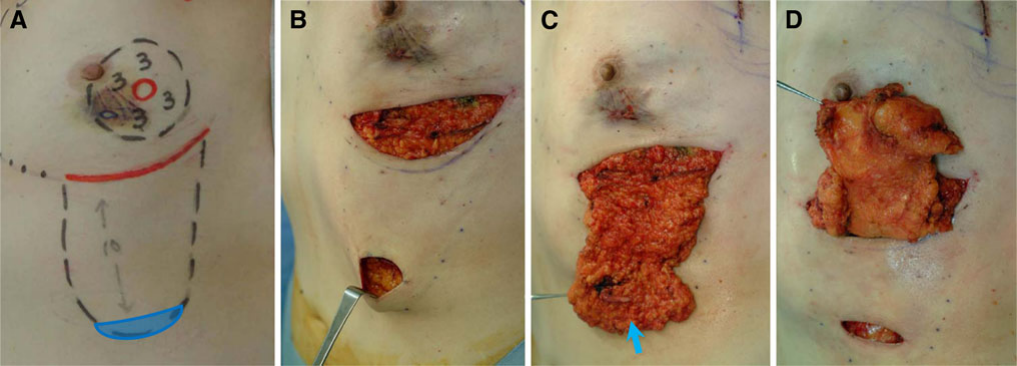
Operative findings. **a** A tongue-shaped adipofascial flap, 10 cm in length, was drawn as a *black dotted line*. A crescent of skin was de-epithelialized. **b** The flap was harvested via an inframammary incision line and a caudal window. **c** The de-epithelialized skin was harvested together with the inframammary flap. **d** The tissue was rolled up towards the cranial side

**Fig. 3:**
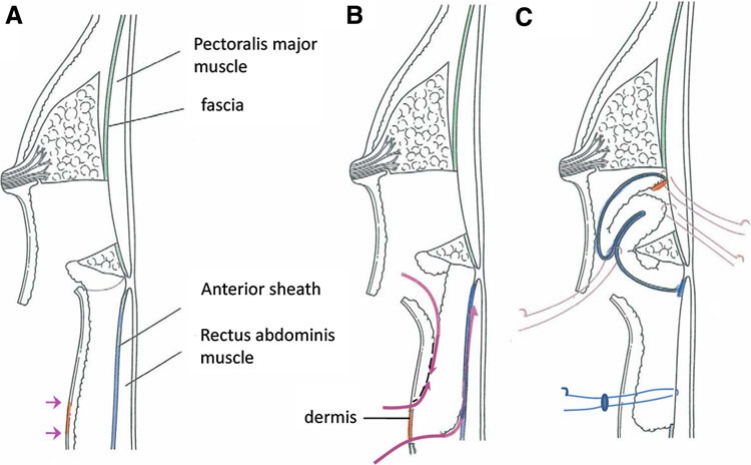
Scheme of the modified inframammary adipofascial flap. **a** A crescent area on the cranial edge of the flap was de-epithelialized. **b** A flap containing the anterior rectus sheath of the abdominis muscle was harvested via two incisions. **c** The flap was placed into the cranial breast cavity and gathered. Anchor sutures were added to the donor site

The harvesting and implantation of the modified inframammary adipofascial flap involves the following five steps: the creation of a de-epithelialized crescent of skin at the caudal edge of the flap; the harvesting of a tongue-shaped graft of cutaneous fat attached to the fascia of the anterior rectus sheath of the abdominis muscle; the rotation of the cutaneous tissue into the cranial defect; the trimming or gathering of the cutaneous tissue to adjust it to the shape of the contralateral breast; and the fixation of the cutaneous tissue to the edge of the remnant gland (Figs. 2, 3). For our patient, we harvested a modified inframammary flap, 10 cm in length, without any special instruments (Fig. 2c).

By using this method, a large amount of fat can be obtained, even if the patient is not obese. Attaching the sheath of the muscle to the adipose tissue makes the harvested tissue firm. It should then be rolled or gathered to reconstruct the breast mound and adjust it so that it is of similar size to the contralateral breast. Fixation to the edge of the remnant gland can then be performed using Vicryl sutures. Several Anchor sutures are added to the donor site using bolsters and kept in place for 3 days after the operation to prevent a seroma forming (Fig. 3c). A continuous closed suction drain is inserted into the subcutaneous defect at the donor site and the reconstructed breast, and left in situ for several days after the operation. In our institution, postoperative radiotherapy is delivered to patients with lesions located within 10 mm of the surgical margin, that contain both invasive and intraductal components. Our patient did not require radiotherapy as histological margins of over 10 mm were maintained from the edge of the resected area to the cancer lesion on permanent sections. Her pathological diagnosis was T1N0M0 according to the TNM classification. She received adjuvant hormone therapy, comprising an aromatase inhibitor (Femara 2.5 mg/day), and has been recurrence-free for 2 years postoperatively.

**Fig. 4:**
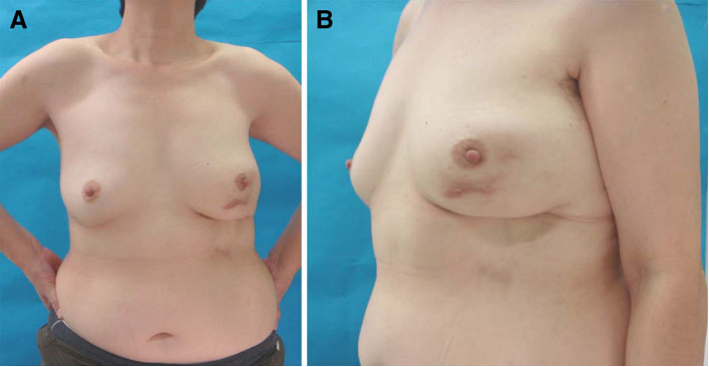
Photograph taken 2 years after the procedure. Although, there was a hypertrophic scar from the CNB scar, symmetry was clearly achieved

Figure 4 summarizes the findings during 2 years of follow-up. We noted slight pigmentation of the skin at the center of the treated area. Now, the volume and size of the patient’s bilateral breasts are symmetric, as are the inframammary line, the bottom of the lowest breast mound, and the nipple area. The deformity at the donor site is inconspicuous anteriorly, especially from the patient’s view.

## Discussion

Postquadrantectomy deformities include localized skin and glandular tissue defects, distortion and/or dislocation of the areola, and retraction of the breast tissue. Perhaps the most prominent and common failure in achieving a good esthetic outcome is a lack of breast symmetry [7]. Surgical techniques used to address the conflict between oncological and cosmetic results are classed under the general term of “oncoplastic surgery” [8]: a new surgical approach that allows wide excision but prevents breast deformities by reconstructing large resection defects immediately [9].

The lower pole of the breast was the first recognized high-risk location for deformity [10–13]. Retraction of the skin and downward deviation of the NAC, resulting from tissue excision from the 6 o’clock position, is known as the “bird’s beak” deformity [10, 13]. Existing mammaplasty techniques were initially modified to become oncoplastic techniques for correcting defects caused by surgery for specific tumors such as lower-pole cancer [10, 11, 14]. Numerous oncoplastic techniques have been developed and are gaining attention in the surgical literature. Several authors have described the utilization of an inverted T-mammoplasty technique for filling defects in all quadrants by extensive mobilization of the lower gland [15–17]. Munhoz et al. concluded that the indications for oncoplastic surgery, combining partial mastectomy with a reduction-type operation, include patients with medium- or large-volume breasts, who have small to moderate defects and enough remaining breast tissue to permit reconstruction. They also concluded that the success of the procedure depends on patient selection, coordinated planning, and careful intraoperative management [15]. According to reports from Western countries, for patients with relatively large and ptotic breasts, oncoplastic surgery combining a reduction-type operation and recentralization of the nipple–areola complex produced good results [18, 19]. However, for most Japanese women, who have smaller breasts than many Western women, it may be more appropriate to reshape the breasts by filling them with extra-breast tissue, rather than performing reduction-type oncoplastic techniques [1–3, 20, 21]. Moreover, some patients do not want to undergo oncoplastic surgery involving a combination of partial mastectomy, reduction, and recentralization of the nipple–areola complex, with or without a contralateral operation to achieve better symmetry. We previously reported how immediate breast reconstruction using an adipofascial flap to fill partial defects in the lower breast is useful for such patients. However, the incision made along the inframammary line to remove the lesion is sometimes far from the edge of the flap, making it difficult to obtain sufficient flap material, without special devices such as longer electric knives, endoscopic instruments, or retractors with lighting systems.

We have since modified our technique for attaching de-epithelialized skin by adding an additional incision to make it easier to harvest the flap. A nutrient vessel of this adipofascial flap is the inframammary intercostal artery perforator [22]. The tiny but certain increase of volume in the adipofascial flap can be improved by attaching dermis. It is unclear if the attached dermis improves blood flow, re-vascularity, and the survival of the flap versus flaps harvested in the standard manner, but it does make the procedures easier. We have no firm results of randomized prospective studies on reconstruction in this area with an inframammary adipofascial flap with or without peripheral dermis. Further study is required to resolve this issue. We did not observe any necrosis or flap volume loss by using either the original or modified technique (Fig. 4). Pigmentation remained in the center of the area from which tissue was removed in this patient, which was attributed to the resection of the scar produced by the CNB. However, postoperative ultrasonography and mammography showed no cystic formation or abnormal calcification.

One of the drawbacks of this technique is the additional scar in the middle of the abdomen; however, this scar is inconspicuous from the patient’s view and there has been no report of sensory or motor abnormalities. Studies on a large number of patients are needed to establish who should receive postoperative radiotherapy after breast-conserving surgery with immediate volume replacement using this flap so that it is acceptable for BCT.

We are aware that longer follow-up is needed for more accurate physical and cosmetic assessment. Based on our limited observation of a small number of patients, we concluded that this procedure provides excellent cosmetic results when performed by a breast surgeon. Oncoplastic surgery, combining partial mastectomy and immediate volume replacement with a modified inframammary adipofascial flap at the time of BCT in the lower region of the breast, can be performed easily and safely with good cosmetic results in patients for whom reduction-type oncoplastic surgery is contraindicated.
